# The Beat

**DOI:** 10.1289/ehp.120-a459

**Published:** 2012-12-03

**Authors:** Erin E. Dooley

## European Acrylamide Levels Largely Unchanged

The European Commission requires the European Food Safety Authority (EFSA) to monitor levels of acrylamide in food products. In its fourth annual assessment of acrylamide in European foods, EFSA reported that although levels decreased in a few food categories between 2007 and 2010, other categories showed increases, with up to 20% of samples in some categories exceeding the values recommended by the European Commission.^^1^^ This chemical, formed in foods during high-temperature cooking, is classified as reasonably anticipated to be a human carcinogen by the National Toxicology Program^^2^^ and has been associated in humans with reduced birth weight and head circumference.^^3^^

**Figure:**
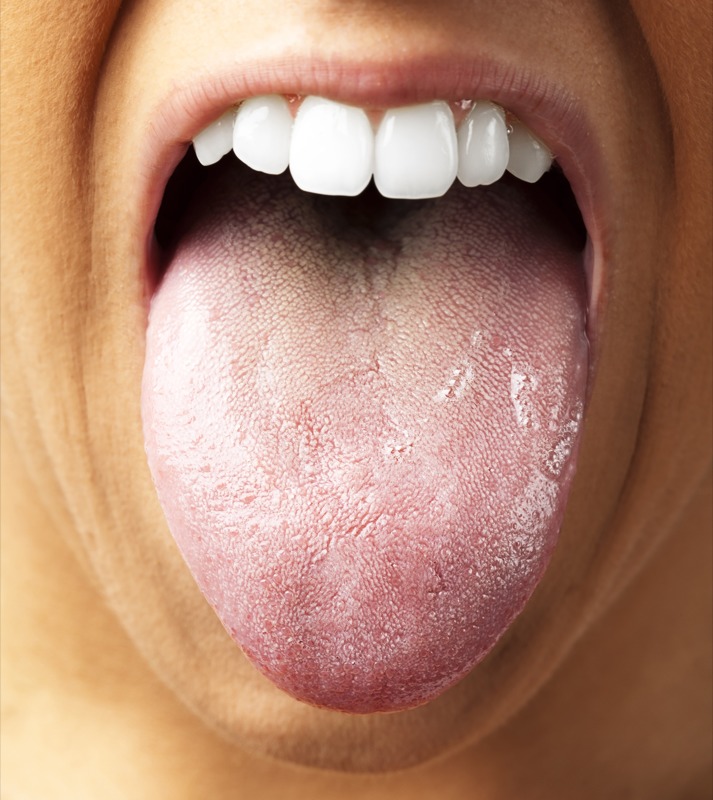
Fried foods are typically high in acrylamide. Shutterstock.com

## Characterizing the Oral Microbiome

A study comparing the bacterial communities in the mouths of fraternal and identical twins found that oral bacteria are largely determined by a person’s environment, rather than by their genes.^^4^^ Comparing oral bacteria between individuals showed that bacterial populations in saliva were less complex than those in the gut, and that twins who cohabitated were more likely to share bacterial communities while living together than after moving apart. The majority of oral bacteria generally fell into one of eight main groups, although each individual carried multiple rare species. These data may be helpful in future studies comparing differences between bacterial populations in healthy and disease states.

**Figure:**
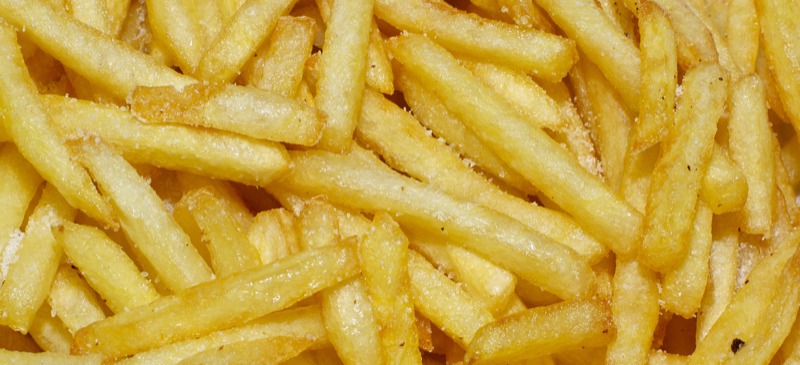
The teeth, gums, tongue, and other parts of the oral cavity provide unique habitats for bacterial colonies. Shutterstock.com

## Impacts of Open-Fire Cooking

Open cooking fires, used by an estimated 3 billion people worldwide, are a major source of pollutants including carbon monoxide, particulate matter, and greenhouse gases. To better understand how open-fire cooking affects regional air quality and associated disease, the National Center for Atmospheric Research has launched a three-year study based in northern Ghana.^^5^^ Team members will combine air quality measurements (gathered in part using smartphones) and computer models of weather, air quality, and climate. Villagers will be surveyed on their beliefs about the connection between open-fire cooking and health effects and their willingness to adopt new cooking methods.

## Mapping Malaria’s Spread in Kenya

People infected with malaria can disperse malarial parasites more widely than mosquitoes can on their own. In a new study, investigators compared cell phone usage data from nearly 15 million Kenyans against detailed malaria incidence maps to study relationships between travel patterns and the spread of malaria.^^6^^ They concluded that malaria largely originated around Lake Victoria and spread southeast to the capital of Nairobi. Using the information gained from this type of analysis may help public health officials target programs for controlling imported cases of malaria. Worldwide in 2010 there were an estimated 216 million cases of malaria and 655,000 deaths, most of them among children under 5 years of age.^^7^^

## FTC Revises Green Guides

In October 2012 the Federal Trade Commission issued its revised Green Guides, the agency’s tool for marketers and consumers regarding green product marketing.^^8^^ The new guides, developed with consumer and industry input, include several new sections covering green certifications and seals of approval, carbon offsets, and claims that products are free of undesirable chemicals, nontoxic, degradable, or made with renewable energy or materials. This is the third revision to the Green Guides, which were first issued in 1992.

**Figure:**
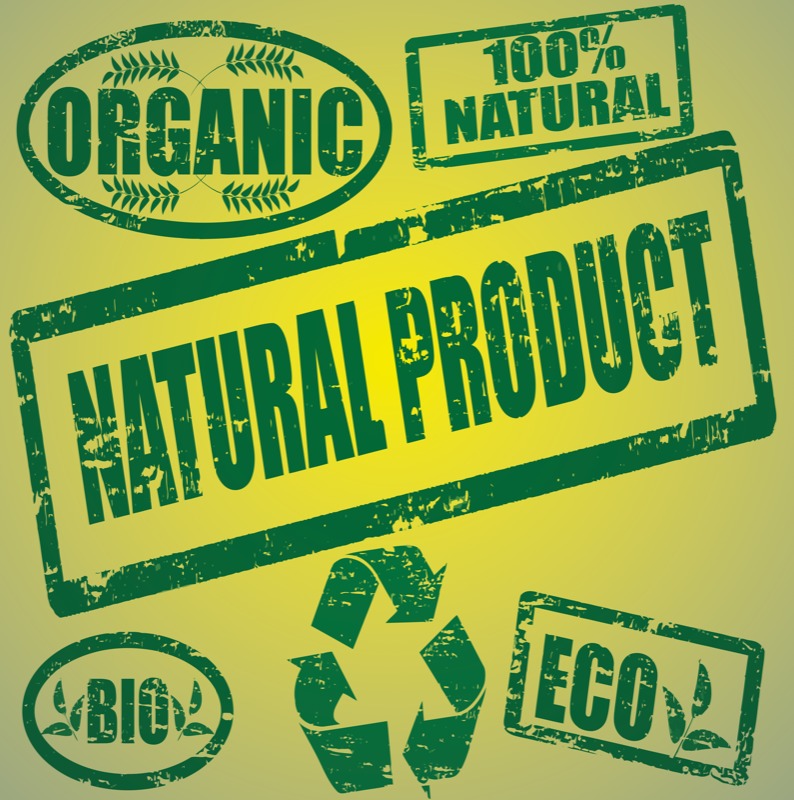
Aaron Amat/Shutterstock.com
